# Chemical, Physical, and Mechanical Properties of 95-Year-Old Concrete Built-In Arch Bridge

**DOI:** 10.3390/ma14010020

**Published:** 2020-12-23

**Authors:** Andrzej Ambroziak, Elżbieta Haustein, Maciej Niedostatkiewicz

**Affiliations:** Faculty of Civil and Environmental Engineering, Gdansk University of Technology, 11/12 Gabriela Narutowicza Street, 80-233 Gdańsk, Poland; elzbieta.haustein@pg.edu.pl (E.H.); mniedost@pg.edu.pl (M.N.)

**Keywords:** structural concrete, reinforced concrete, bridge engineering, material characterization, mechanical properties, chemical properties

## Abstract

This research aimed to determine the durability and strength of an old concrete built-in arch bridge based on selected mechanical, physical, and chemical properties of the concrete. The bridge was erected in 1925 and is located in Jagodnik (northern Poland). Cylindrical specimens were taken from the side ribs connected to the top plate using a concrete core borehole diamond drill machine. The properties of the old concrete were compared with the present and previous standard requirements and guidelines. The laboratory testing program consisted of the following set of tests: measurements of the depth of carbonated zone and dry density, water absorption tests, determination of concrete compressive strength and frost resistance, determination of modulus of elasticity, measurement of the pH value, determination of water-soluble chloride salt and sulfate ion content, and X-ray diffraction analyses. Large variations in the cylindrical compressive strength (14.9 to 22.0 MPa), modulus of elasticity (17,900 to 26,483 MPa), density (2064 to 2231 kg/m^3^), and water absorption (3.88 to 6.58%) were observed. In addition to the experiments, a brief literature survey relating to old concrete properties was also conducted. This paper can provide scientists, engineers, and designers an experimental basis in the field of old concrete built-in bridge construction.

## 1. Introduction

Since the second half of the 19th century when reinforced concrete was invented, there has been a rapid development of this composite material, which is made up of a combination of steel and concrete [1]. Reinforced concrete is a material used in the construction of a wide range of civil and engineering structures. Due to concrete degradation, high traffic, and the impact of high load, old concrete and reinforced concrete bridges or other types of structures require improvement, repair, and reconstruction. Before taking any action and starting the design process, it is necessary to form an expert opinion by carrying out a detailed examination and laboratory tests of the construction materials used in the old structure. In several cases, it is necessary to incorporate the scientific and engineering community to evaluate the performance of old structures. In the literature, it is possible to find many interesting descriptions related to the process of testing and repairing old concrete structures. Hellebois et al. [2,3] performed an investigation on hardened concrete samples removed from a narrow-gauge railway viaduct (Colo-Hugues viaduct) built in Belgium in 1904. Mechanical and durability performance of the 100-year-old hardened concrete samples were found to be remarkably good. Sena-Cruz et al. [4] described historical, geometrical, and damage surveys of a reinforced concrete bridge built in 1907 (the Luiz Bandeira bridge). Selected structural material properties were also determined, e.g., the strength class was found to be greater than C30/37 and the average modulus of elasticity was 30 GPa. Wolert et al. [5] investigated an 11-span flat slab reinforced concrete bridge constructed between 1914 and 1916 that goes over Barnes Slough and Jenkins Creek in the USA. The authors confirmed the overall good condition of the structure and its reserve flexural capacity. Onysyk et al. [6] described the strength of the reinforced concrete ribbed dome of Centennial Hall in Wrocław, Poland, which was built in 1911–1913. Gebauer and Harni [7] examined the composition and microstructure of the hydrated cement paste of an 84-year-old reinforced concrete bridge construction. Tests showed that the main hydration products were calcium hydroxide, fibrous calcium silicate hydrate incorporated frequently with calcium hydroxide into hexagonal plates, and calcium aluminate carbonate hydrate. Qazweeni and Daoud [8] studied the physical, mechanical, and chemical properties of a 20-year-old concrete structure in an office building. Blanco et al. [9] investigated the chemical reactions leading to the degradation of a 95-year-old concrete dam manufactured with sand–cement as a binder. Ambroziak et al. [10] determined the durability and strength of reinforced concrete continuous footing based on selected mechanical and chemical properties of a 70-year-old concrete structure in an office building. Melchers et al. [11] performed observations and analysis on a 63-year-old reinforced concrete promenade railing exposed to the harsh sea-spray environment of the North Sea in Arbroath, Scotland. Castro-Borges et al. [12] studied the physical and mechanical properties of a 60-year-old concrete pier with stainless steel reinforcement. The pier showed no visible sign of deterioration after 60 years of service. Sohail et al. [13] investigated the effects of concrete degradation in structural concrete elements in reinforced concrete structures built in the 1960s, 1970s, and 1980s in the Arabian Gulf region. The carbonation depth and chloride concentration profiles were determined from concrete core samples. Papé and Melchers [14] performed load tests on full-scale prestressed beams sampled from the 45-year-old Sorell Causeway bridge in Tasmania, Australia. The prestressing strands showed severe localized corrosion with cross-section losses between 75% and 100%. Dasar et al. [15] tested 40-year-old reinforced concrete beams exposed to real marine environments for up to 20 years at Sakata Port, Japan. The deterioration and performance reduction were investigated, and a good correlation was observed between the crack width and cross-section loss. Czaderski and Motavalli [16] performed experimental investigations on a full-scale concrete bridge girder strengthened with prestressed carbon fiber-reinforced polymer (CFRP) plates obtained from the Viadotto delle Cantine a Capolago bridge, which was constructed in 1964–1966. Usage of the gradient method for anchorage of prestressed CFRP plates on large-scale girders was confirmed. Pettigrew et al. [17] carried out experiments on 48-year-old concrete bridge girders fabricated using lightweight concrete after the decommissioning of a bridge in the USA. The designed flexural capacities were overestimated by an average of 34.0% compared to the values measured in laboratory tests. Khan et al. [18] tested reinforced concrete beams corroded by 26 years of exposure to a chloride environment. The corrosion had a significant impact on the load-carrying capacity, stiffness, and deflection of the beams. Prassianakis and Giokas [19] determined the mechanical properties of 28-year-old concrete using destructive and ultrasonic nondestructive testing methods. Chen [20] studied the dynamic mechanical properties of 10-year-old concrete exposed to high temperatures. Zhu et al. [21] studied the durability and mechanical properties of a 10-year-old crumb rubber concrete bridge deck. The investigation concluded that the deck was in good condition. Kou and Poon [22] investigated the mechanical properties of five-year-old concrete prepared with 0, 20, 50, and 100% recycled aggregates used as replacements of natural aggregates. Dasar et al. [23] studied the applicability of seawater as a mixing and curing agent in four-year-old mortar cement. The laboratory tests indicated that the effect of seawater on corrosion activity was considerably higher as a curing agent than as a mixing agent. Many engineering and scientific studies investigating the mechanical, chemical, and/or physical properties of built structures take into consideration the subject of old concrete. A proper assessment of the properties of old concrete helps determine the range of repair or reconstruction required as well as the load capacity of the investigated structure, which is needed for ensuring extended working life and the safe use of old facilities.

The present study aimed to determine selected mechanical, physical, and chemical properties of a 95-year-old concrete arch bridge. Cylindrical specimens were taken from the side ribs connected to the top plate using a concrete core borehole diamond drill machine. The drilling locations were selected based on their availability and limited interference to the bridge structure. The investigation presented in this study can be treated as part of expert opinion on the bearing capacity of the arch bridge in order to determine the possibility of the bridge carrying additional loads and help extend its working life. This paper provides scientists, engineers, and designers an experimental assessment of the mechanical, physical, and chemical properties of 95-year-old concrete.

## 2. Materials and Methods

**Figure 1:**
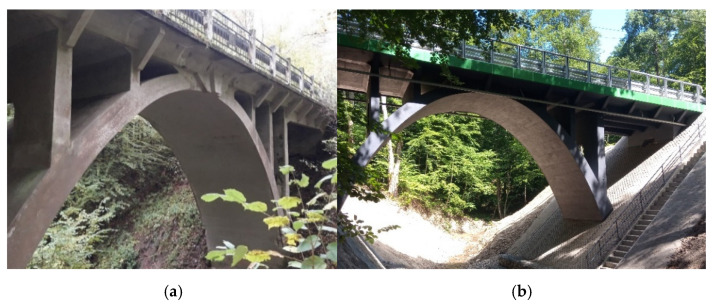
Jagodnik arch bridge before (**a**) and after (**b**) reconstruction.

The arch bridge investigated in this study is located above a forest canyon and the Kumiel river (watercourse) (see Figure 1). The structure of the center span bridge (about 12.95 m) is a reinforced concrete slab, monolithically connected to reinforced concrete shields supported on a plate arch. The arch bridge was built in 1925 in Jagodnik (Poland) by Karl Metzger & Co. building company (see p. 181 in [24], where a photo of the investigated arch bridge is inserted). In 1925, Jagodnik (Berendshagen) was part of the district of Elbing in Germany (present-day Elbląg in Poland). The arch bridge was erected under the guidelines of the German Committee for Structural Concrete issued in January 1916 [25] for structural use of concrete, design, and construction. This guideline was in force until September 1925, when the German standard DIN 1045 [26] was introduced.

Laboratory tests were carried out to determine the chemical, physical, and mechanical properties of old concrete. For this purpose, concrete specimens were taken from structural elements of the bridge, namely the side ribs connected to the top plate, using a concrete core borehole diamond drill machine. The thickness of the reinforced concrete side ribs was approximately 15 cm. Cylindrical specimens were taken from five different side ribs connected to the top plate using a concrete core borehole diamond drill machine. The spacing between the side ribs was approximately 2 m. The samples were marked as location number_specimen number (e.g., 1_2, 5_1, etc.). These denotations were used for all laboratory tests.

Two types of cylindrical samples were prepared for mechanical tests from the exploratory bore holes:

- Type 1 had a length to core diameter ratio *L*/*D* = 1, with diameter *D* of approximately 100 mm (*f*_is,cycl 100_); 19 samples were used for uniaxial compressive tests.
- Type 2 had a length to core diameter ratio *L*/*D* = 1.5, with diameter *D* of approximately 100 mm; 7 samples were used to measure the modulus of elasticity.

The dimensions of the concrete cores for concrete compressive strength tests were determined according to the standard EN 12504-1 [27], with the preferred length/diameter ratio of 1.0. The strength results determined for the concrete cores *f*_c,cycl 100_ were comparable to the cube strength *f*_c,cube_ of 15 × 15 × 15 cm concrete specimens (i.e., *f*_c,cube_ = *f*_c,cycl 100_). The ASTM C469M standard [28] states that the ratio between the specimen length *L* and the dimension *D* should be greater than 1.50; thus, the concrete cores for the determination of the modulus of elasticity was taken as *L*/*D* = 1.5. In laboratory tests, the application of a greater diameter and/or greater length to core diameter ratio (e.g., *L*/*D* = 2 as in the ASTM C31 standard [29]) is often impossible for old concrete structures [10].

Selected chemical, physical, and mechanical properties of the 95-year-old concrete built-in arch bridge were investigated using laboratory tests. The laboratory testing program consisted of the following sets of tests.

### 2.1. Measurements of the Depth of Carbonated Zone

The depth of the carbonated zone was measured with phenolphthalein solution. Freshly fractured surfaces of old concrete were submitted under an alcoholic solution of phenolphthalein, which immediately reacted, turning to pink/purple color, indicating the presence of calcium hydroxide, except at the thin, already carbonated external layers.

### 2.2. Measurements of Dry Density

The method specified in the EN 12390-7 standard [30] was applied for determining the density of the 95-year-old concrete. The tested specimens were dried in a ventilated oven at 105 ± 5 °C until the mass changed by less than 0.2%. Before weighing, each specimen was cooled to near room temperature in a dry, airtight vessel.

### 2.3. Tests of Water Absorption

Water absorption tests were carried out following EN 13369, Annex G [31]. To measure the water uptake capacity of concrete samples, the specimens were soaked in drinking water to a constant mass and then oven-dried in a ventilated drying oven at 105 ± 5 °C to a constant mass.

### 2.4. Determination of Concrete Compressive Strength and Frost Resistance

Uniaxial compressive tests were undertaken using a computer-controlled mechanical testing machine with a constant rate of loading and a range of 0.6 MPa/s according to the EN 12390-3 standard [32]. The frost resistance of the old concrete was determined according to guidelines given by the PN-B-06250 standard [33]. A freezing chamber with a temperature- and time-controlled refrigerating and heating system was used. The freezer cycle consisted of freezing at −18 ± 2 °C for 4 h and thawing by total immersion in water at 18 ± 2 °C for 4 h.

### 2.5. Determination of Modulus of Elasticity

The ASTM C469M standard [28] guideline was used to determine the modulus of elasticity. Diamond-drilled concrete cores with a length to diameter ratio of 1.50 were used in a compressometer device to measure the static modulus of elasticity.

### 2.6. Measurement of the pH Value and Determination of Water-Soluble Chloride Salts (Cl^−^) and Sulfate Ions (SO_4_^2−^)

The pH was measured according to ISO 10523 [34]. The extract with water-soluble sulfate ions and chloride ions were specified according to EN 1744-1 + A1 standard [35]. The extract with chloride ions was determined in accordance with the Volhard method. The concentration of water-soluble chloride salts and sulfate ions as well as the pH of the test samples were measured after dissolving a given amount of mass of crushed concrete in distilled water. After filtration through a mixed cellulose ester (MCE) membrane filter with a pore size of 45 μm, the obtained filtrates were tested.

### 2.7. X-ray Diffraction (XRD) Analyses

The microstructure of the cross sections of samples was studied using a JEOL JSM-7800F (Akishima, Tokyo, Japan) scanning electron microscope (SEM) equipped with an energy-dispersive X-ray spectrometer (EDAX, Octane Elite, Mahwah, NJ, USA), which allowed the element composition of the tested samples to be identified. The acceleration voltage in the X-ray tube for surface analysis of samples was 15 kV. The X-ray beam current was 5 nm. Observations were carried out to identify different phases of the microstructure.

## 3. Results and Discussion

### 3.1. Measurements of Depth of Carbonated Zone

**Figure 2:**
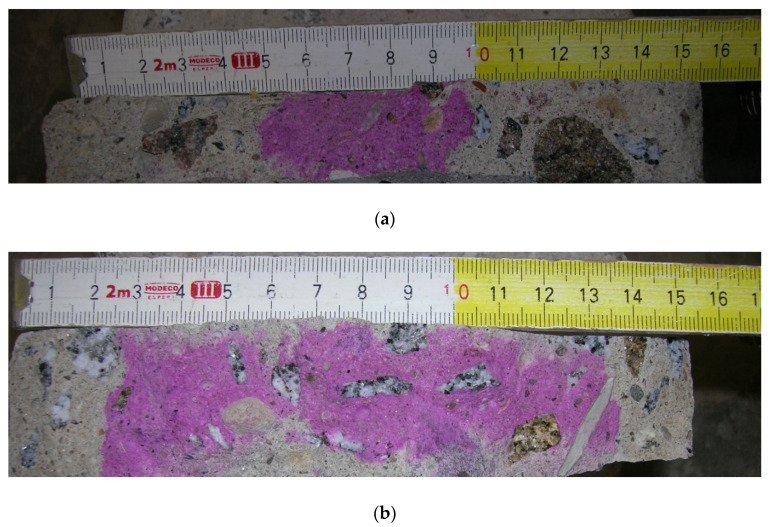
Depth of carbonated zone: (**a**) about 5 cm; (**b**) about 2.5 cm.

The chemical reaction of calcium hydroxide dissolved in pore water with atmospheric carbon dioxide (conversion into calcium carbonate, which is then mainly calcite [36]) is called carbonation of concrete. Carbon dioxide in the air penetrates into concrete and diminishes the pH value and also causes shrinkage in the concrete [37]. The depth of the carbonated zone measured with the phenolphthalein solution is illustrated in Figure 2. Freshly fractured surfaces of all specimens reacted with the alcoholic solution of phenolphthalein, immediately turning to pink/purple color, indicating the presence of calcium hydroxide, except at the thin, already carbonated external layers. Large variations in depth of the carbonated zone was observed in the investigated specimens of old concrete, ranging approximately 20 to 55 mm (see Table 1). The average depth of carbonation of the old concrete was 36 ± 2 mm. The result of the mean value is presented as the sum of mean values and standard error of the mean of the specified range.

### 3.2. Measurements of Dry Density and Water Absorption Tests

The dry density is one of the important parameters determined for concrete. Concrete is a mixture, and its density depends on its ingredients and their proportions. The mean dry density value specified in laboratory tests was 2175 ± 7 kg/m^3^ (see Table 2). The dry density ranged from 2000 to 2600 kg/m^3^; thus, according to the EN 206 standard [38], the investigated old concrete could be categorized as normal concrete. The mean dry density value also fulfilled conditions for normal-weight concrete according to the ACI 318-19 standard [39] (density between 2160 and 2560 kg/m^3^).

The laboratory tests determined water absorption of the old concrete as ranging from 3.88 to 6.58% (see Table 2). The mean value of water absorption was 5.84 ± 0.11%. According to the PN-88/B-06250 standard [33] guidelines, water absorption should not be greater than 5% for concrete exposed to atmospheric conditions and not greater than 9% for concrete protected from atmospheric conditions. PN-S-10040 [40] states that the water absorption of concrete used in bridge structures should not be greater than 5%. The mean water absorption values in our study were greater than 5%; thus, according to the International Federation for Structural Concrete (fib) report [41], the concrete quality could be categorized as poor quality.

### 3.3. Concrete Compressive Strength, Frost Resistance, and Modulus of Elasticity

**Figure 3:**
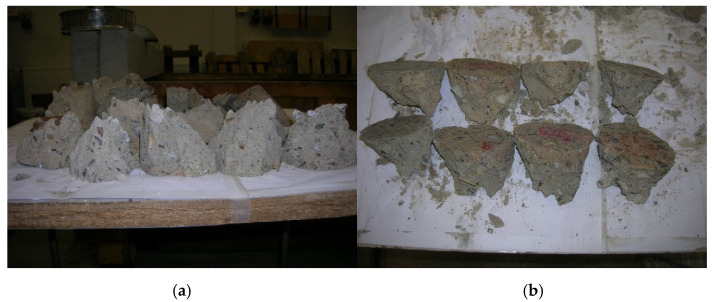
Concrete specimens after uniaxial compressive tests: (**a**,**b**) views of the form of failure.

Uniaxial compressive experimental tests were carried out using the Advantest 9 C300KN mechanical testing machine. Experiments were performed on the failure of the concrete cylinder specimens (see Figure 3). The uniaxial tensile test results of compressive strength for cylindrical samples *f*_c,cycl 100_ is presented in Table 3. The mean value of compressive strength of cylindrical samples *f*_c,cycl 100_ was 18.8 ± 0.7 MPa. The strength results of the cylindrical samples *f*_c,cycl 100_ with length/diameter ratio of 1.0 were comparable to the cube strength *f*_c,cube_ of 15 × 15 × 15 cm concrete specimens according to the EN 12504-1 standard [27], i.e., *f*_c,cycl 100_ = *f*_c,cube_ = 18.8 ± 0.7 MPa. The variation in compressive strength values of the 95-year-old concrete (see Table 3) can be explained by the production technology, which was probably based on portable concrete mixers with handmade proportions of concrete components. Portland cement was used as a binder in ordinary old concrete mixes. It should be noted that the new cementitious materials, e.g., geopolymer concrete (called alkali-activated materials, see e.g., [42,43,44,45,46]), have higher compressive strength and better durability compared to concrete mixes containing Portland cement. 

**Figure 4:**
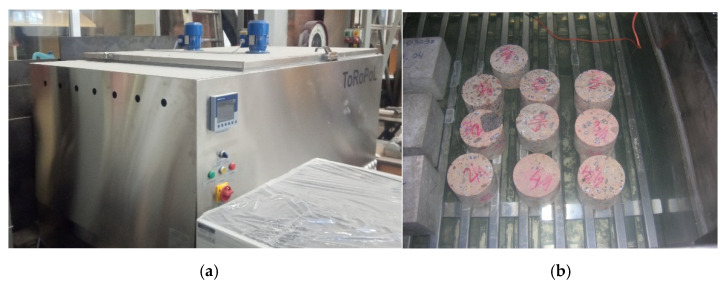
Frost resistance tests: (**a**) view of freezing chamber; (**b**) view of concrete specimens inside the freezing chamber.

With regard to evaluation of freezing resistance, according to the PN-B-06250 standard [33], the compressive strength should not decrease by more than 20% in comparison to the base samples and the specimens should not show cracks [47]. In this study, we started the test by saturating the concrete samples with water. Then, 10 concrete samples were placed in a freezing chamber with a temperature- and time-controlled refrigerating and heating system (see Figure 4). The concrete samples were placed in the freezer compartment with a minimum 20 mm gap. A total of 50 freezer cycles were carried out consisting of freezing at −18 ± 2 °C for 4 h and thawing by total immersion in water at 18 ± 2 °C for 4 h. After the last defrosting, a strength test was carried out. The frost resistance assessment was based on measuring the change in compressive strength. The mean value of compressive strength of the cylindrical samples after 50 freezer cycles $f_{c,\mathrm{cycl}\;100}^{50\;\mathrm{freezer}\;\mathrm{cycles}}$ was 17.9 ± 1.0 MPa, which was about 5% lower than the compressive strength of cylindrical samples *f*_c,cycl 100_ without freezing (base samples). The difference between compressive strength *f*_c,cycl 100_ and $f_{c,\mathrm{cycl}\;100}^{50\;\mathrm{freezer}\;\mathrm{cycles}}$ was small (<20% according to PN-B-06250 [33]); therefore, it could be stated that the 95-year-old concrete possessed freezing resistance. 

The two moduli of elasticity of applicable customary working stress ranged from 0 to 40% (*E*_0.0–0.4_) and from 10 to 30% (*E*_0.1–0.3_) were specified. Seven cylindrical specimens with a length/diameter ratio of 1.5 were stored and tested at room temperature (approximately 20 °C) in air-dry conditions. According to the laboratory tests, the modulus of elasticity ranged from 17.9 to 27 GPa for *E*_0.0–0.4_ and from 20 to 27 GPa for *E*_0.1–0.3_ (see Table 4). The mean values of the modulus of elasticity were 22,890 ± 1320 MPa for *E*_0.0–0.4_ and 22,730 ± 890 MPa for *E*_0.1–0.3_. The difference between the mean values of modulus of elasticity for *E*_0.0–0.4_ and *E*_0.1–0.3_ was very small (less than 1%). It should be noted that the EN 1992-1-1 standard [48] defines the modulus of elasticity as a secant value of 0 to 40% of the ultimate strength for concrete with quartzite aggregates. A limit of 10–30% should be used for limestone and sandstone aggregates. The ASTM C469M standard [28] also indicates 40% ultimate load to calculate modulus of elasticity. On the other hand, 30% of the ultimate strength is required in the ISO 1920-10 standard [49].

Hallauer [50] indicated that Hennebique seems to recommend a mixture consisting either of 1 part cement, 2 parts sand, and 4 parts gravel or 1 part cement, 3 parts sand, and 5 parts gravel (aggregate mix: sand: 0/7 mm, gravel: 7/70 mm, stone grit: 7/25 mm, stone chip: 25/70). Forecast compressive strength was about 15–18 MPa after 28 days and 18–24.5 MPa after 45 days. Note that the decrease in strength with the same cement content was due to the addition of water. The old Polish PN-B-195 standard [51] specified concrete strength equal to 0 (zero) MPa to emphasize that the amount of water should be limited and controlled in the concrete mix. The present guidelines on concrete standards state the requirements for water to cement ratio without mentioning zero-strength concrete. Wolert et al. [5] obtained compressive test results varying from 12.1 to 23.0 MPa for core samples cut out from an 11-span flat slab reinforced concrete bridge constructed between 1914 and 1916. Our laboratory tests determined the mean compressive strength of the 95-year-old concrete built-in arch bridge as *f*_c,cycl 100_ = 18.8 MPa, which is similar to concrete structures build during this time period. The 95-year-old concrete also had frost resistance (after 50 freezer cycles, the compressive strength decreased only 5% to $f_{c,\mathrm{cycl}\;100}^{50\;\mathrm{freezer}\;\mathrm{cycles}}$ = 17.9 MPa). However, the compressive strength of old concrete construction sometimes varies and exhibits a wide range of compressive strengths [10]. Hellebois and Espion [3] investigated 107-year-old concrete samples taken from the Colo-Hugues viaduct, which was designed and built in 1904 in Belgium, and found different compressive strengths: 54.2 MPa for the slab, 33.3 MPa for beams, and 19.7 MPa for the column. Variation in the compressive strength of concrete is related to the type of aggregates and cement applied in concrete mixes [52] and is also affected by environmental conditions during the placement process of concrete mixes [53].

The characteristic in-situ compressive cube strength *f*_ck,is,cube_ can be estimated according to the EN 13791 standard [54] as follows:

(1) $$f_{\mathrm{ck},\mathrm{is},\mathrm{cube}}=\min\left\{\begin{array}{c} f_{m(n),\mathrm{is}}-k_{n}\cdot s\\ f_{\mathrm{is},\mathrm{lowest}}-M \end{array}\right\}=\min\left\{\begin{array}{c} 18.8-1.96\cdot 0.7\\ 14.9+2 \end{array}\right\}=\min\left\{\begin{array}{c} 17.43\\ 16.9 \end{array}\right\}=16.9\;\mathrm{MPa}$$

where *f*_m(n),is_ is the mean in-situ compressive strength of *n* test results (in the present investigation, *n* = 9), *f*_is,lowest_ is the lowest in-situ compressive strength test results, *k*_n_ is the factor that depends on the number of test results (*k*_n_ = 1.96 for *n* = 9 test results, see Table 6 in EN 13791 [54]), *s* is the standard deviation of in-situ compressive strength, and *M* is the value of margin (*M* = 2, see Table 7 in the EN 13791 standard [54]). The characteristic in-situ cube compressive strength *f*_ck,is,cube_ = 16.9 MPa; thus, the C12/15 compressive strength class according to the EN 206 [38] and EN 13791 [54] standards could be specified. The secant modulus of elasticity for the C12/15 concrete strength class specified in the EN 1992-2 standard [48] is 27,000 MPa. The mean modulus of elasticity *E*_0.0–0.4_ determined by laboratory tests (see Table 4) was 22,890 MPa, which was about 15% lower than that specified in the EN 1992-2 standard [48] for design of new concrete structures. However, it should be noted that the modulus of elasticity depends not only on the strength class but also on the types and properties of the aggregates used to prepare concrete mixes. For the design of new concrete bridge structures, the EN 1992-2 standard [55] recommends the application of minimum strength classes not less than C30/37. According to the EN 206 standard [38], the C30/37 compressive strength class should have a minimum characteristic cylinder strength (*f*_ck,cyl_) of 30 MPa (N/mm^2^) and cube strength (*f*_ck,cube_) of 37 MPa. The Polish standard PN-S-10042 [56] states that the C20/25 strength class may be applied for new foundations, supports, and retaining walls where the dimension of the structural elements are not less than 0.6 m thick, while the C25/30 strength class may be applied for new elements of supports and retaining walls less than 0.6 m thick and for reinforced concrete spans.

### 3.4. Chemical Properties

The pH value and the content of water-soluble chloride salts (Cl^−^) and sulfate ions (SO_4_^2−^) were determined using a chemical testing program. Samples for chemical analysis were taken from the cover layer (denotated as 1s, 2s, 3s, 4s, and 5s) and center layer (denotated as 1c, 2c, 3c, 4c, and 5c) of the 95-year-old concrete samples. The cover layer samples for chemical investigations were obtained by cutting approximately 2–2.5 cm cylindrical samples from the exploratory boreholes.

The mean value of the pH was 10.6 ± 0.4 for the cover layer (range from 9.47 to 11.68) and 12.29 ± 0.04 for the center layer of samples (range from 12.14 to 12.37) (see Table 5 and Table 6). The pH value of the concrete pore solution decreased as carbonation proceeded. The pH value for freshly made concrete varies from 12.5 to 13.5 [57]. The pH value is one of the most useful factors (influencing the corrosion rate [58]) for specifying the ability of concrete to protect steel rebar by the formation of a passive film protecting reinforcement from corrosion [59]. The corrosion of rebar generally occurs when the pH value is less than 9 (the passive film is not stable, see e.g., [60]). Despite the large depth of the carbonated zone (see Table 1), the pH of the old concrete was still in the safety range.

The content of water-soluble chloride salts and sulfate ions was determined as a percentage of dry weight (see Table 5 and Table 6). The water-soluble chloride salt values ranged from 0.012 to 0.036% dry weight for the cover layer and from 0.010 to 0.018% dry weight for the center layer of samples. The mean values of water-soluble chloride salt were 0.019 ± 0.004% and 0.014 ± 0.001% for the cover layer and the center layer, respectively. The sulfate ion values ranged from 0.127 to 0.670% dry weight for the cover layer and from 0.011 to 0.089% dry weight for the center layer of samples. The mean values of sulfate ions were 0.39 ± 0.11% and 0.031 ± 0.014% for the cover layer and the center layer, respectively.

In 1925, the cement content was usually set at 300 kg/m^3^; it could be 270 kg/m^3^ for buildings without the influence of moisture (see e.g., [50]). Assuming usage of 300 kg/m^3^ cement and specified dry density of 2175 kg/m^3^ (see Table 2), it was possible to convert the percentage content of chloride ions and sulfate ions by mass of cement (see Table 7). The chloride content of the concrete samples expressed as a percentage of chloride ions by mass of cement did not exceed 0.2 and 0.15%, which are limits for reinforced concrete stated by the EN 206 standard [38] and the ACI 318 code [61], respectively. The 95-year-old concrete built-in arch bridge was not exposed to chloride attack. A chloride content in concrete of over 0.2–0.3% of cement weight can be treated as being exposed to chloride attack.

The mean percentage content of sulfate ions SO_4_^2−^ by mass of cement was 2.83 for the cover layer and 0.22 for the center layer of concrete samples (see Table 7). The concrete samples did not exceed 4%, which is the limit by mass of cement based on the total acid-soluble sulfate given by the BS 8110-1:1985 standard [62] (this guideline was excluded in the new edition of the BS 8110-1 standard [63]). The low concentration of sulfate ions in the concrete samples indicated that the low contamination was due to external sources.

### 3.5. X-ray Diffraction Analyses

**Figure 5:**
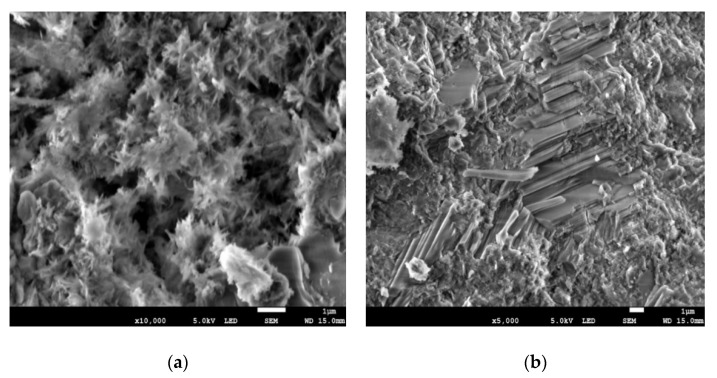
Scanning electron microscope (SEM) images of the samples taken from the cover layer of the concrete structure: (**a**) ×10,000; (**b**) ×5000.

**Figure 6:**
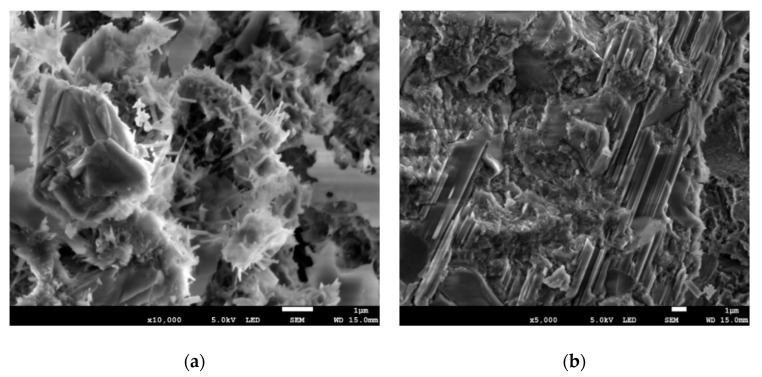
SEM images of the samples taken from center layer of the concrete structure: (**a**) ×10,000; (**b**) ×5000.

Based on the specified chemical properties, samples from 1s and 1c concrete specimens were chosen for XRD analyses to assess the presence of crystalline compounds and identify them. For the tests, specimens measuring 10 × 10 × 10 mm were taken from the cover layer and the center layer (about 7.5 cm below the surface of the concrete) of the tested concrete element. Microstructure tests were carried out for four samples (two samples from the cover and two samples from the center layer of old concrete specimens). Element composition tests (EDS) were carried out for four samples (three samples from the cover and three samples from a depth of about 7.5 cm below the surface). The results of the microstructure (SEM images) and element composition of the tested samples are shown in Figure 5 and Figure 6.

**Figure 7:**
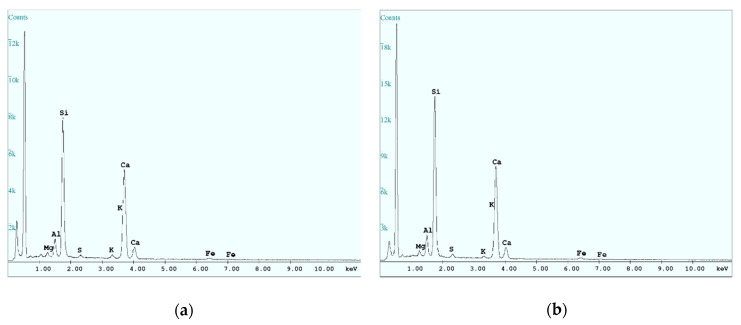
Energy-dispersion X-ray spectrometer (EDS) spectra of samples taken from the (**a**) cover layer; (**b**) center layer.

The results of element composition tests are presented in Table 8 and Table 9. They indicated the dominance of two main elements in the microstructure of the tested samples: Si (silica) and Ca (calcium) (see Figure 7). The content of these elements for samples taken from the cover layer was 29.4 to 34.7% for silica and 54.1 to 57.5% for calcium. For the specimen samples taken from center layer, the content was 25.6 to 36.8% for silica and 51.6 to 60% for calcium. The content of other elements, such as Al, Fe, Mg, K, and Na, was definitely lower compared to the Si and Ca content in all samples.

## 4. Conclusions

The main objective of the present research was to determine the durability and strength of a 95 year-old concrete built-in arch bridge in Jagodnik (northern Poland) based on selected mechanical, physical, and chemical properties. Based on the experimental investigation, the following conclusions can be drawn:

- The old concrete could be categorized as normal concrete according to the EN 206 standard [38], with a mean dry density value of 2175 ± 7 kg/m^3^ (see Table 2).
- The average depth of carbonation of the old concrete was 36 ± 2 mm (see Table 1). The old concrete had large variations in depth of the carbonated zone ranging from 20 to 55 mm. Despite the large depth of the carbonated zone, the pH of the old concrete was still in the safety range.
- The mean values of water absorption was 5.84 ± 0.11% (see Table 2), so the quality of the old concrete could be categorized as poor quality.
- The mean value of the compressive strength of cylindrical samples *f*_c,cycl 100_ was 18.8 ± 0.7 MPa. Estimated characteristic in-situ compressive cube strength *f*_ck,is,cube_ was 16.9 MPa (according to the EN 13791 standard [54]). The old concrete was categorized as C12/15 compressive strength class according to EN 206 [38]. However, the 95-year-old concrete possessed good freezing resistance.
- The mean value of the modulus of elasticity *E*_0.0–0.4_ (see Table 4) was 22,890 MPa, which was about 15% lower than that specified in the EN 1992-2 standard [48] for C12/15 concrete strength class.
- The mean value of the pH was 10.6 ± 0.4 and 12.29 ± 0.04 for the cover layer and the center layer of samples, respectively (see Table 5 and Table 6). The pH values for the old concrete indicated that there was no corrosion of the steel rebars. Generally, when the pH value decreases below 9–9.5, corrosion of the reinforcing steel may be indicated.
- The chloride content of the old concrete did not exceed 0.2% by mass of cement; thus, the old concrete arch bridge was not exposed to chloride attack. The low concentration of sulfate ions in the old concrete samples indicated that the low contamination was due to external sources.
- The element composition tests indicated the dominance of two main elements (Si and Ca) in the microstructure of the old concrete (see Table 8 and Table 9). The significant content of the two elements suggested complete reaction of the clinker phases of the cement over the time the structure of concrete was exposed to the environment.
- Portlandite crystals were found in the tested samples, shaped most often as hexagonal platelets. Both isolated and clustered occurrence was noted. The visible portlandite occurred in the form of large pseudohexagonal crystals, forming columnar conglomerates arranged in parallel. In both cases, the resulting products (phases CSH and CH) were due to cement hydration reactions with water. The microstructure of the concrete has a significant influence on its properties, such as its strength and durability.

The design team of the Jagodnik arch bridge made a decision on the possibility of reconstructing the 95-year-old arch bridge and designing it for car traffic to a limited tonnage. On top of the existing old structure, a new deck slab with pavement covers was made. The entire surface of the old concrete was protected with repair mortars.

Deterioration of old concrete due to corrosion is a serious problem. Proper assessment of the properties of old concrete is critical to make decisions regarding the reconstruction and repair of old concrete structures. Inappropriate or incorrect estimation of the properties of old concrete can have catastrophic consequences. Reconstruction and renovation of old civil and building structures often require incorporation of the scientific and engineering community in order to evaluate the performance of old structures and give them “new life” and extended service. The authors are hopeful that this investigation sparks interest among a wide group of engineers and scientists to take into consideration the subject of old concrete structures.

## Figures and Tables

**Table 1:** Measurements of depth of carbonated zone.

Specimen No.	1_1	1_2	2_1	2_2	3_1	3_2	4_1	4_2	5_1	5_2	Mean (mm)
Cover 1 (mm)	45	35	40	44	30	40	50	30	25	24	36 ± 2
Cover 2 (mm)	45	35	40	45	25	40	55	32	25	20	

**Table 2:** Dry density and water absorption.

Specimens No.	Dry Density (kg/m^3^)	Water Absorption (%)
1_1	2166	6.29
1_2	2183	6.01
1_3	2171	6.05
1_4	2212	5.68
1_5	2157	6.12
1_6	2186	6.02
1_7	2207	5.85
2_1	2162	5.94
2_2	2230	5.69
2_3	2132	6.53
2_4	2173	5.93
3_1	2154	6.47
3_2	2184	6.05
3_3	2155	5.58
3_4	2180	5.88
3_5	2170	5.84
3_6	2193	5.80
3_7	2179	5.02
4_1	2162	6.14
4_2	2064	5.98
4_3	2180	5.69
4_4	2231	5.35
5_1	2229	3.88
5_2	2174	5.27
5_3	2147	6.58
5_4	2191	6.25
Mean	2175 ± 7	5.84 ± 0.11

**Table 3:** Concrete compressive strength.

Specimens No.	Compressive Strength *f*_c,cycl 100_ (MPa)	Specimens No.	Compressive Strength after 50 Freezer Cycles (MPa)
1_2	14.9	1_1	12.7
1_3	20.0	1_4	17.0
2_3	19.1	2_1	20.3
3_2	18.6	2_2	18.7
3_3	22.0	3_1	18.4
4_2	19.9	3_4	13.9
4_3	19.5	4_1	16.3
5_2	16.1	4_4	19.1
5_3	18.9	5_1	22.6
		5_4	20.3
Mean	18.8 ± 0.7	Mean	17.9 ± 1.0

**Table 4:** Modulus of elasticity.

Specimens No.	*E*_0.0–0.4_ (MPa)	*E*_0.1–0.3_ (MPa)
1_5	22,567	21,095
1_6	20,613	21,643
1_7	26,483	24,497
2_4	25,369	22,832
3_5	20,264	22,026
3_6	27,027	27,027
3_7	17,900	20,006
Mean	22,890 ± 1320	22,730 ± 890

**Table 5:** pH values of concrete specimens and content of chloride ions and sulfate ions in concrete as a percentage of dry weight for the cover layer.

Samples	pH	Cl^−^ (%)	SO_4_^2−^ (%)
1s	9.47	0.036	0.670
2s	9.98	0.020	0.298
3s	10.54	0.014	0.658
4s	11.37	0.014	0.127
5s	11.68	0.012	0.203
Mean	10.6 ± 0.4	0.019 ± 0.004	0.39 ± 0.11

**Table 6:** pH values of concrete specimens and content of chloride ions and sulfate ions in concrete as a percentage of dry weight for the center layer.

Samples	pH	Cl^−^ (%)	SO_4_^2−^ (%)
1c	12.32	0.017	0.089
2c	12.37	0.018	0.019
3c	12.14	0.014	0.022
4c	12.28	0.013	0.011
5c	12.34	0.010	0.016
Mean	12.29 ± 0.04	0.014 ± 0.001	0.031 ± 0.014

**Table 7:** Mean content of chloride ions and sulfate ions in concrete as a percentage of mass of cement.

Layer	Cl^−^ (%)	SO_4_^2−^ (%)
Cover	0.14	2.83
Center	0.10	0.22

**Table 8:** Element compositions of samples taken from the cover layer of the concrete structure as determined by EDS.

Type of Element	Sample 1	Sample 2	Sample 3	Mean (%)
Mg	1.2	1.0	1.4	1.2
Al	4.8	5.0	6.2	5.3
Si	36.8	34.7	25.6	32.4
S	1.3	1.3	1.8	1.5
K	1.0	1.6	1.0	1.2
Na	-	0.6	-	0.6
Ca	51.7	51.6	60.0	54.4
Fe	3.3	3.3	3.9	3.5
F	-	1.0	-	1.0

**Table 9:** Element compositions of samples taken from the center layer of the concrete structure as determined by EDS.

Type of Element	Sample 1	Sample 2	Sample 3	Mean (%)
Mg	1.1	1.3	1.2	1.2
Al	4.3	5.3	5.7	5.1
Si	34.7	28.9	29.4	31.0
S	1.1	0.8	1.0	0.9
K	1.7	1.7	1.7	1.7
Ca	54.1	58.0	57.5	56.6
Fe	3.2	3.4	3.5	3.4

## Data Availability

All laboratory test results (data) are included in Table 1, Table 2, Table 3, Table 4, Table 5, Table 6, Table 7, Table 8 and Table 9 in the present paper. No new data were created or analyzed in this study. Data sharing is not applicable to this article.
